# Impacts of new sex- and age-specific TSH and FT4 reference ranges on thyroid status and metabolic disorder associations

**DOI:** 10.1210/jendso/bvag053

**Published:** 2026-03-10

**Authors:** Sayaka Yamada, Kazuhiko Horiguchi, Masako Akuzawa, Koji Sakamaki, Eijiro Yamada, Tetsuro Andou, Masanobu Yamada

**Affiliations:** Department of Internal Medicine, Division of Endocrinology and Metabolism, Gunma University Graduate School of Medicine, Maebashi, Gunma 3718511, Japan; Department of Internal Medicine, Division of Endocrinology and Metabolism, Gunma University Graduate School of Medicine, Maebashi, Gunma 3718511, Japan; Hidaka Hospital, Takasaki, Gunma 3700001, Japan; Hidaka Hospital, Takasaki, Gunma 3700001, Japan; Department of Internal Medicine, Division of Endocrinology and Metabolism, Gunma University Graduate School of Medicine, Maebashi, Gunma 3718511, Japan; Hidaka Hospital, Takasaki, Gunma 3700001, Japan; Department of Internal Medicine, Division of Endocrinology and Metabolism, Gunma University Graduate School of Medicine, Maebashi, Gunma 3718511, Japan; Thyroid Federation Japan, Tokyo 1050023, Japan

**Keywords:** reference ranges, free-thyroxine, thyroid-stimulating hormone, thyroid status, metabolic disorders

## Abstract

**Context:**

Thyroid hormone levels vary by age, sex, and genetic background; yet most clinical reference ranges (RRs) do not account for these factors, potentially leading to misclassification of thyroid status and missed associations with metabolic disorders.

**Objective:**

The aim of this study was to evaluate the impact of newly established age- and sex-specific reference ranges for thyroid-stimulating hormone (TSH) and free thyroxine (FT4) on the prevalence of thyroid statuses and their associations with metabolic disorders.

**Methods:**

Cross-sectional observational studies were conducted, and we enrolled over 8000 participants who underwent thyroid status evaluation with Abbott and Siemens assay kits. We compared the prevalence of different thyroid statuses and corresponding metabolic disorders based on the new RRs with that based on the manufacturers’ RRs.

**Results:**

New RRs significantly altered the prevalence of several thyroid statuses. Subclinical hypothyroidism increased in middle-aged individuals using Abbott kits but decreased in older individuals using Siemens kits. Syndrome of inappropriate secretion of TSH (SITSH, high FT4 with normal or elevated TSH) increased significantly with both kits when applying the new RRs. SITSH, defined by the new RRs, was significantly associated with increased risks of hypertension (Abbott: odds ratio [OR] 2.00; Siemens: OR 1.70) and diabetes (Abbott: OR 2.65; Siemens: OR 1.68), whereas no such associations were observed using the manufacturers’ RRs.

**Conclusion:**

Age- and sex-specific RRs corrected the misclassification of thyroid status and uncovered a previously underdiagnosed group (SITSH) associated with cardiometabolic risks. These findings support the importance of new RRs for diagnostic accuracy and clinical risk stratification.

In clinical practice, thyroid function is evaluated by measuring serum thyroid-stimulating hormone (TSH) and free-thyroxine (FT4) levels because serum free-triiodothyronine (FT3) levels are generally unreliable and difficult to measure due to their low concentration and limited accuracy of immunoassays [1, 2]. The levels of TSH and FT4 are typically measured using immunoassays and evaluated using consistent manufacturers’ recommended reference ranges (RRs), without considering sex and age.

In this way, abnormal thyroid statuses are diagnosed, such as overt hyperthyroidism (high FT4/low TSH), hypothyroidism (low FT4/high TSH), and other thyroid statuses, including the syndrome of inappropriate secretion of TSH (SITSH; high FT4/with normal or slightly high TSH), which is widely used in Japan and refers to a condition closely related to what is termed “inappropriate secretion of TSH” [3, 4], and individuals with low FT4 but normal/low TSH. Subsequently, various examinations are conducted to determine whether abnormal thyroid function is secondary to systemic illness, medication, or physiological variation, or due to primary thyroid disorders. Additionally, since serum TSH levels are highly sensitive to slight changes in serum thyroid hormone (TH) levels, even when within normal ranges, subclinical thyroid dysfunctions can be diagnosed. Subclinical hypothyroidism is diagnosed by the presence of slightly elevated serum TSH levels, above the upper reference range and sometimes exceeding 10 mIU/L, with TH and particularly FT4 levels within the normal RR [5]. Conversely, subclinical hyperthyroidism can be diagnosed when serum TSH levels are reduced, below the lower reference range and sometimes undetectable, but TH levels remain within normal ranges [6]. Thus, TSH and FT4 are helpful for diagnosing the majority of thyroid disorders.

Manufacturers’ recommended RRs have been determined using assays of a few hundred healthy individuals, and the middle 95% levels (levels within 1.96 standard deviations of the mean) have typically been used, as the middle 95% of a well-defined and sufficiently large disease-free population is likely to provide the most appropriate balance between true and false diagnoses [7, 8]. Thyroid status evaluation depends on the upper and lower limits of serum TSH and FT4 RR levels. In cases of subclinical thyroid dysfunction, diagnoses are made based only on these RRs, without including clinical manifestations [9]. Furthermore, previous studies suggest that these RRs may differ based on sex and age [10-13].

In most previous studies, the prevalence and associated complications of subclinical thyroid dysfunction and the effectiveness of its treatment have been investigated using the assay manufacturers’ RRs [10, 14-21]. Some results have been inconsistent, particularly for older individuals [22-27], possibly because RRs vary with age in healthy individuals.

We recently reported changes in serum TSH and TH levels according to age and sex, based on assays from three different manufacturers and a relatively large Japanese cohort [28]. We demonstrated age-dependent increases in serum TSH levels in both men and women, with higher levels in women than in men of the same age. Serum FT4 levels were higher in men, particularly in young men, than in women, and decreased with age only in men. FT3 levels showed similar changes to FT4 levels.

Thus, abnormal thyroid status prevalence is likely to change when using age- and sex-appropriate RRs. We examined more than 8000 participants using two different assay kits to compare the prevalence of thyroid status defined using either the manufacturers’ RRs or age- and sex-specific RRs. In addition, we investigated the risk of metabolic disorders associated with thyroid function according to each set of RRs.

## Materials and methods

**Figure 1:**
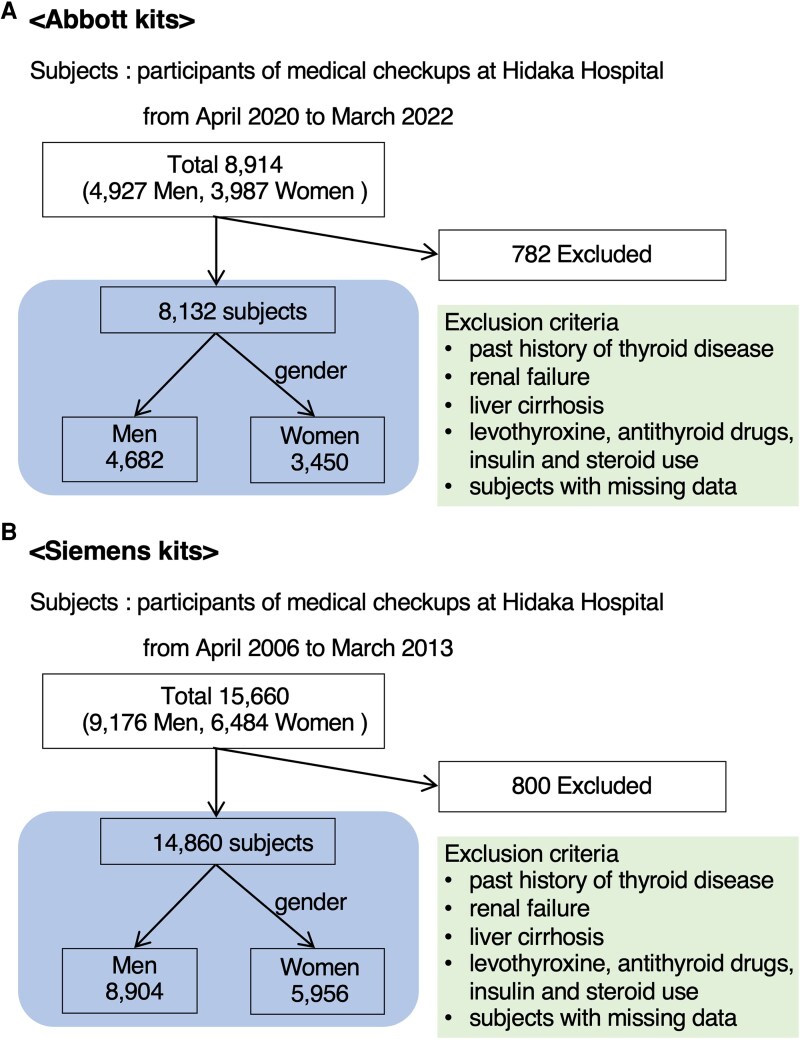
Flow diagrams for participants assessed using Abbott kits (A: upper panel) and using Siemens kits (B: lower panel).

Among individuals who underwent health checkups as in- or outpatients at Takasaki Hidaka Hospital (Takasaki, Japan), 8132 (4682 men and 3450 women) were tested using Abbott kits (Chicago, IL) between 2020 and 2022, and 14 860 (8904 men, 5956 women) were tested using Siemens kits (Munich, Germany) between 2006 and 2013 (Fig. 1).

Participants completed a self-administered questionnaire with items on medical history and medication use. Patients with any history of thyroid disease, liver cirrhosis, or renal failure; those using medications that affect TH levels, including levothyroxine, antithyroid drugs, insulin, and steroid hormones; and those with missing data were excluded.

Previously reported age- and sex-specific RRs for serum TSH and TH levels were used to define thyroid statuses [28]. Overt hyperthyroidism and hypothyroidism were defined as high serum FT4/low TSH levels and low serum FT4/high TSH levels, respectively. Subclinical hypothyroidism was defined as elevated serum TSH/normal FT4 levels. Subclinical hyperthyroidism was defined as reduced serum TSH/normal FT4 levels. SITSH was defined as high FT4 and normal/elevated serum TSH. Low FT4 levels and normal/reduced serum TSH levels are often indicative of central hypothyroidism.

Metabolic disorders were defined as follows: Dyslipidemia was defined as a serum low-density lipoprotein cholesterol level of 140 mg/dL or higher, a triglyceride level of 150 mg/dL or higher, a high-density lipoprotein cholesterol level less than 40 mg/dL [29], or a documented history of dyslipidemia or current use of lipid-lowering medication. Hypertension was defined as a documented history of hypertension or current use of antihypertensive medication. Diabetes mellitus was defined as a fasting blood glucose level of 126 mg/dL or higher, an HbA1c value of 6.5% or higher, or a documented history of diabetes or current use of antidiabetic medication.

The study was conducted according to the principles of the Declaration of Helsinki and the relevant guidelines and regulations, including the Ethical Guidelines for Medical and Health Research Involving Human Subjects presented by the Ministry of Health, Labor, and Welfare in Japan. Approval was obtained from the Ethics Committee on Human Research of Gunma University Hospital (approval number HS2022-055). According to the Ministry guidelines, written informed consent was not required for this study design. We disclosed the outline of our study and provided opportunities for disagreement and withdrawal at any time during the investigation.

### Blood tests for serum TH and TSH levels

Blood samples were collected from patients between 08:00 and 09:00, after fasting for at least 11 hours. Serum TSH and TH levels were measured using an Architect i2000SR in the hospital laboratory, using the following kits:

Architect TSH CLIA and FT4•Abbott CLIA (Abbott, Inc.) were used to measure TSH and FT4, respectively. The manufacturers’ RRs were 0.35-4.94 mIU/L (99th percentile), 0.70-1.48 ng/dL (99th percentile) for TSH and FT4, respectively.

Additionally, Chemilumi ACS II, CLEIA, Chemilumi E-FT4, and CLEIA (Siemens Healthcare Diagnostics) were used to measure TSH and FT4 levels, respectively. The RRs established by Hidaka Hospital were 0.4-4.0 mIU/L (wider than the 95th percentile but less than the 99th percentile) and 0.8-1.9 ng/dL (wider than the 95th percentile but less than the 99th percentile) for serum TSH and FT4, respectively.

The kits were switched from Siemens to Abbott due to an institutional change. As measurement values may vary depending on the kits, the use of two kits enabled us to determine whether the observed results reflected kit-specific variation or represented a consistent pattern across different kits.

### Statistical analyses

Mean, standard deviation, and *P*-value were calculated using JMP 15.2.0 statistical software (SAS Institute Inc., Cary, NC, USA). In calculating the percentage of thyroid status across decades to make the total 100%, the proportion of individuals with normal thyroid function was adjusted because this group constituted the majority and was least influenced by numerical adjustments. Comparisons of prevalence based on age- and sex-specific RRs or manufacturers’ RRs were conducted using McNemar's test. Odds ratios (ORs) and 95% confidence intervals (CIs) were calculated using logistic regression analysis adjusted for age and sex. Statistical significance was set at a *P*-value of less than .05.

## Results

### Participant characteristics

Figure 1 depicts a flow diagram of the participants measured using the two kits. Overall, 8914 and 15 660 participants were examined using the Abbott and Siemens kits, respectively. We excluded 782 (245 men and 537 women) and 800 (272 men and 528 women) patients and examined 4682 men and 3450 women and 8904 men and 5956 women with the two kits, respectively. The mean ages of the men and women evaluated using the Abbott and Siemens kits were 54.05 ± 11.20 (range, 23-88) and 51.74 ± 10.84 (range, 22-86) years, and 49.67 ± 9.93 (range, 20-88) and 48.53 ± 10.16 (range, 19-88) years, respectively. The number of participants in each decade for the two kits is shown in the tables (Tables 1-4).

**Table 1:** Thyroid statuses based on FT4 and TSH using the manufacturers’ reference ranges or the age- and sex-specific reference ranges with Abbott kits in women

Thyroid statuses based on FT4 and TSH using the manufacturers' reference ranges with Abbott kits in women										
	normal	subclinical hypothyroidism	subclinical hyperthyroidism	overt hypothyroidism	overt hyperthyroidism	TSH high FT4 high	TSH normal FT4 high	TSH normal FT4 low	TSH low FT4 low	total
**−19**	0	0	0	0	0	0	0	0	0	0
**20**-**29**	22 (91.66)	1 (4.17)	1 (4.17)	0	0	0	0	0	0	24
**30**-**39**	443 (97.80)	3 (0.66)	6 (1.32)	0	1 (0.22)	0	0	0	0	453
**40**-**49**	1009 (97.49)	9 (0.87)	13 (1.26)	0	2 (0.19)	0	0	2 (0.19)	0	1035
**50**-**59**	1082 (97.48)	15 (1.35)	11 (0.99)	0	0	0	1 (0.09)	1 (0.09)	0	1110
**60**-**69**	593 (95.96)	16 (2.59)	8 (1.29)	0	1 (0.16)	0	0	0	0	618
**70**-**79**	182 (93.81)	10 (5.15)	1 (0.52)	0	0	0	0	1 (0.52)	0	194
**80**-**89**	13 (92.86)	0	0	1 (7.14)	0	0	0	0	0	14
**total(%)**	3344 (96.97)	54 (1.57)	40 (1.16)	1 (0.03)	4 (0.12)	0	1 (0.03)	4 (0.12)	0	3448

Thyroid statuses based on FT4 and TSH using the age- and sex-specific reference ranges with Abbott kits in women										
	normal	subclinical hypothyroidism	subclinical hyperthyroidism	overt hypothyroidism	overt hyperthyroidism	TSH high FT4 high	TSH normal FT4 high	TSH normal FT4 low	TSH low FT4 low	total
**−19**	0	0	0	0	0	0	0	0	0	0
**20**-**29**	24 (100)	0	0	0	0	0	0	0	0	24
**30**-**39**	416 (91.82)	10 (2.21)	7 (1.55)	1 (0.22)	2 (0.44)	0	8 (1.77)	9 (1.99)	0	453
**40**-**49**	950 (91.79)	21 (2.03)	17 (1.64)	3 (0.29)	5 (0.48)	1 (0.10)	18 (1.74)	20 (1.93)	0	1035
**50**-**59**	1018 (91.72)	23 (2.07)	24 (2.16)	4 (0.36)	3 (0.27)	0	23 (2.07)	15 (1.35)	0	1110
**60**-**69**	565 (91.44)	13 (2.10)	13 (2.10)	2 (0.32)	1 (0.16)	0	13 (2.10)	11 (1.78)	0	618
**70**-**79**	182 (93.80)	3 (1.55)	2 (1.03)	1 (0.52)	0	0	3 (1.55)	2 (1.03)	1 (0.52)	194
**80**-**89**	14 (100)	0	0	0	0	0	0	0	0	14
**total(%)**	3169 (91.90)	70 (2.03)	63 (1.83)	11 (0.32)	11 (0.32)	1 (0.03)	65 (1.89)	57 (1.65)	1 (0.03)	3448

**Table 2:** Thyroid statuses based on FT4 and TSH using the manufacturers’ reference ranges or the age- and sex-specific reference ranges with Abbott kits in men

Thyroid statuses based on FT4 and TSH using the manufacturers' reference ranges with Abbott kits in men										
	normal	subclinical hypothyroidism	subclinical hyperthyroidism	overt hypothyroidism	overt hyperthyroidism	TSH high FT4 high	TSH normal FT4 high	TSH normal FT4 low	TSH low FT4 low	total
**−19**	0	0	0	0	0	0	0	0	0	0
**20**-**29**	19 (95.00)	0	1 (5.00)	0	0	0	0	0	0	20
**30**-**39**	466 (98.52)	0	6 (1.27)	0	0	0	1 (0.21)	0	0	473
**40**-**49**	1159 (97.90)	5 (0.42)	19 (1.60)	0	1 (0.08)	0	0	0	0	1184
**50**-**59**	1462 (97.39)	17 (1.13)	19 (1.27)	1 (0.07)	1 (0.07)	0	0	1 (0.07)	0	1501
**60**-**69**	1030 (96.90)	20 (1.88)	12 (1.13)	0	1 (0.09)	0	0	0	0	1063
**70**-**79**	396 (95.89)	16 (3.87)	1 (0.24)	0	0	0	0	0	0	413
**80**-**89**	27 (96.43)	1 (3.57)	0	0	0	0	0	0	0	28
**total(%)**	4559 (97.38)	59 (1.26)	58 (1.24)	1 (0.02)	3 (0.06)	0	1 (0.02)	1 (0.02)	0	4682

Thyroid statuses based on FT4 and TSH using the age- and sex-specific reference ranges with Abbott kits in men										
	normal	subclinical hypothyroidism	subclinical hyperthyroidism	overt hypothyroidism	overt hyperthyroidism	TSH high FT4 high	TSH normal FT4 high	TSH normal FT4 low	TSH low FT4 low	total
**−19**	0	0	0	0	0	0	0	0	0	0
**20**-**29**	20 (100)	0	0	0	0	0	0	0	0	20
**30**-**39**	434 (91.77)	9 (1.90)	11 (2.32)	0	0	1 (0.21)	8 (1.69)	10 (2.11)	0	473
**40**-**49**	1081 (91.32)	28 (2.36)	25 (2.11)	1 (0.08)	3 (0.25)	0	23 (1.94)	23 (1.94)	0	1184
**50**-**59**	1369 (91.20)	34 (2.27)	31 (2.07)	3 (0.20)	3 (0.20)	0	29 (1.93)	32 (2.13)	0	1501
**60**-**69**	975 (91.71)	24 (2.26)	21 (1.98)	2 (0.19)	2 (0.19)	0	19 (1.79)	20 (1.88)	0	1063
**70**-**79**	381 (92.26)	9 (2.18)	10 (2.42)	1 (0.24)	0	0	7 (1.69)	5 (1.21)	0	413
**80**-**89**	28 (100)	0	0	0	0	0	0	0	0	28
**total(%)**	4288 (91.59)	104 (2.22)	98 (2.09)	7 (0.15)	8 (0.17)	1 (0.02)	86 (1.84)	90 (1.92)	0	4682

**Table 3:** Thyroid statuses based on FT4 and TSH using the manufacturers’ reference ranges or the age- and sex-specific reference ranges with Siemens kits in women

Thyroid statuses based on FT4 and TSH using the manufacturers' reference ranges with Siemens kits in women										
	normal	subclinical hypothyroidism	subclinical hyperthyroidism	overt hypothyroidism	overt hyperthyroidism	TSH high FT4 high	TSH normal FT4 high	TSH normal FT4 low	TSH low FT4 low	total
**−19**	1 (100)	0	0	0	0	0	0	0	0	1
**20**-**29**	55 (84.61)	7 (10.77)	2 (3.08)	0	1 (1.54)	0	0	0	0	65
**30**-**39**	1083 (93.94)	52 (4.51)	14 (1.21)	2 (0.17)	2 (0.17)	0	0	0	0	1153
**40**-**49**	1934 (93.97)	98 (4.76)	16 (0.78)	2 (0.10)	5 (0.24)	0	0	2 (0.10)	1 (0.05)	2058
**50**-**59**	1684 (93.14)	103 (5.70)	15 (0.83)	4 (0.22)	2 (0.11)	0	0	0	0	1808
**60**-**69**	636 (87.47)	83 (11.42)	6 (0.83)	0	2 (0.28)	0	0	0	0	727
**70**-**79**	109 (87.20)	13 (10.4)	1 (0.80)	0	2 (1.60)	0	0	0	0	125
**80**-**89**	16 (84.21)	2 (10.53)	0	1 (5.26)	0	0	0	0	0	19
**total(%)**	5518 (92.64)	358 (6.01)	54 (0.91)	9 (0.15)	14 (0.24)	0	0	2 (0.03)	1 (0.02)	5956
										

Thyroid statuses based on FT4 and TSH using the age- and sex-specific reference ranges with Siemens kits in women										
	normal	subclinical hypothyroidism	subclinical hyperthyroidism	overt hypothyroidism	overt hyperthyroidism	TSH high FT4 high	TSH normal FT4 high	TSH normal FT4 low	TSH low FT4 low	total
**−19**	1 (100)	0	0	0	0	0	0	0	0	1
**20**-**29**	63 (96.92)	1 (1.54)	0	0	0	0	1 (1.54)	0	0	65
**30**-**39**	1067 (92.54)	25 (2.17)	24 (2.08)	2 (0.17)	3 (0.26)	0	25 (2.17)	7 (0.61)	0	1153
**40**-**49**	1925 (93.54)	46 (2.24)	32 (1.55)	4 (0.19)	10 (0.49)	0	29 (1.41)	11 (0.53)	1 (0.05)	2058
**50**-**59**	1689 (93.43)	40 (2.21)	25 (1.38)	4 (0.22)	6 (0.33)	0	31 (1.71)	13 (0.72)	0	1808
**60**-**69**	686 (94.35)	17 (2.34)	14 (1.93)	1 (0.14)	3 (0.41)	0	5 (0.69)	1 (0.14)	0	727
**70**-**79**	120 (96.00)	3 (2.40)	0	0	2 (1.60)	0	0	0	0	125
**80**-**89**	19 (100)	0	0	0	0	0	0	0	0	19
**total(%)**	5570 (93.51)	132 (2.22)	95 (1.60)	11 (0.18)	24 (0.40)	0	91 (1.53)	32 (0.54)	1 (0.02)	5956

**Table 4:** Thyroid statuses based on FT4 and TSH using the manufacturers’ reference ranges or the age- and sex-specific reference ranges with Siemens kits in men

Thyroid statuses based on FT4 and TSH using the manufacturers' reference ranges with Siemens kits in men										
	normal	subclinical hypothyroidism	subclinical hyperthyroidism	overt hypothyroidism	overt hyperthyroidism	TSH high FT4 high	TSH normal FT4 high	TSH normal FT4 low	TSH low FT4 low	total
**−19**	0	0	0	0	0	0	0	0	0	0
**20**-**29**	54 (96.43)	2 (3.57)	0	0	0	0	0	0	0	56
**30**-**39**	1382 (96.85)	24 (1.68)	13 (0.91)	0	4 (0.28)	0	3 (0.21)	0	1 (0.07)	1427
**40**-**49**	2852 (96.02)	75 (2.53)	26 (0.88)	1 (0.03)	10 (0.34)	0	4 (0.13)	2 (0.07)	0	2970
**50**-**59**	2837 (95.22)	111 (3.72)	26 (0.87)	1 (0.03)	1 (0.03)	0	3 (0.10)	1 (0.03)	0	2980
**60**-**69**	1152 (92.23)	86 (6.89)	9 (0.72)	0	1 (0.08)	0	0	1 (0.08)	0	1249
**70**-**79**	174 (86.13)	25 (12.38)	2 (0.99)	1 (0.50)	0	0	0	0	0	202
**80**-**89**	15 (75.00)	5 (25.00)	0	0	0	0	0	0	0	20
**total(%)**	8466 (95.10)	328 (3.68)	76 (0.85)	3 (0.03)	16 (0.18)	0	10 (0.11)	4 (0.04)	1 (0.01)	8904

Thyroid statuses based on FT4 and TSH using the age- and sex-specific reference ranges with Siemens kits in men										
	normal	subclinical hypothyroidism	subclinical hyperthyroidism	overt hypothyroidism	overt hyperthyroidism	TSH high FT4 high	TSH normal FT4 high	TSH normal FT4 low	TSH low FT4 low	total
**−19**	0	0	0	0	0	0	0	0	0	0
**20**-**29**	54 (96.42)	1 (1.79)	0	0	0	0	1 (1.79)	0	0	56
**30**-**39**	1349 (94.54)	32 (2.24)	13 (0.91)	1 (0.07)	4 (0.28)	1 (0.07)	16 (1.12)	10 (0.70)	1 (0.07)	1427
**40**-**49**	2815 (94.76)	70 (2.36)	26 (0.88)	2 (0.07)	10 (0.34)	0	21 (0.71)	26 (0.88)	0	2970
**50**-**59**	2747 (92.19)	66 (2.21)	68 (2.28)	5 (0.17)	3 (0.10)	0	66 (2.21)	25 (0.84)	0	2980
**60**-**69**	1164 (93.20)	26 (2.08)	18 (1.44)	3 (0.24)	1 (0.08)	1 (0.08)	17 (1.36)	19 (1.52)	0	1249
**70**-**79**	194 (96.03)	3 (1.49)	2 (0.99)	1 (0.50)	0	0	2 (0.99)	0	0	202
**80**-**89**	20 (100)	0	0	0	0	0	0	0	0	20
**total(%)**	8343 (93.71)	198 (2.22)	127 (1.43)	12 (0.13)	18 (0.20)	2 (0.02)	123 (1.38)	80 (0.90)	1 (0.01)	8904

### Comparison of euthyroid participants

Using Abbott kits, the percentage of euthyroid participants (with normal serum FT4 and TSH values) defined by the manufacturers’ RRs decreased when evaluated using the new RRs (from approximately 97% to 92% in both women and men) (Tables 1 and 2). In contrast, with the Siemens kit, the percentage was slightly increased (by 1%) in women and decreased slightly (by 1%) in men (Tables 3 and 4).

Next, we examined the number and percentage of euthyroid participants in each decade age group. With the Abbott kits, the number of euthyroid individuals aged 30-79 years decreased when using the new RRs, without sex-specific changes (Tables 1 and 2). With the Siemens kits, the number of euthyroid individuals increased among those older than 60 years when using the new RRs, whereas the numbers were slightly decreased in middle-aged (30-49 years of age) women (Table 3). Similar changes were observed in men (Table 4). Therefore, when compared to Siemens kits, Abbott kits underdiagnosed abnormal thyroid function, particularly in middle-aged women and men. However, Siemens kits overdiagnosed abnormal thyroid function in older individuals when using the manufacturer's RRs.

### Comparison of overt hyper- and hypothyroid statuses

Using Abbott kits, the percentage of overt hyperthyroidism diagnosed with the new RRs increased approximately threefold compared to that using the manufacturers’ RRs, from 0.12% to 0.32% in women and 0.06% to 0.17% in men. The number and percentage of middle-aged women and men defined as having overt hyperthyroidism were significantly higher with the new RRs (*P* = .008 and *P* = .025, respectively; Tables 1 and 2). Similarly, with Siemens kits, in women, the percentage of participants defined as having overt hyperthyroidism (0.24%) using the manufacturers’ recommended RRs nearly doubled to 0.40% with the new RRs, mainly due to the increase in the number of middle-aged women (Table 3). In men, the percentage did not change markedly (0.18% and 0.20%, respectively; Table 4). Strikingly, the prevalence of overt hypothyroidism increased about 10-fold when using the new RRs with Abbott kits (from 0.03% to 0.32%; *P* = .004, and 0.02% to 0.15%; *P* = .014 in women and men, respectively). Specifically, the number and percentage of men and women with overt hypothyroidism in middle age increased significantly (Tables 1 and 2). However, with the Siemens kits, no significant changes in the prevalence of overt hypothyroidism were observed in women between the manufacturers’ RRs and new RRs, although a significant increase was observed in men using the new RRs (*P* = .027).

Overall, the new age-relevant FT4 RR led to approximately 3-10 times higher prevalence of overt hyper- and hypothyroidism in women and men, except for overt hyperthyroidism in men and overt hypothyroidism in women using Siemens kits. Thus, using the current manufacturers’ recommended RRs may lead to underdiagnosis of overt hyper- and hypothyroidism.

### Comparison of subclinical hypo- and hyperthyroidism

The prevalence of subclinical thyroid disorders was determined using Abbott kits (Tables 1 and 2). The prevalence of subclinical hypothyroidism in women was 1.57% with the manufacturers’ RRs and increased slightly to 2.03% using the new RRs. The results were similar in men, with the prevalence of subclinical hypothyroidism being 1.26% and 2.22% when using the manufacturers’ RRs and the new RRs, respectively. These increases were primarily due to an increase in the number of middle-aged individuals. In contrast, using Siemens kits (Tables 3 and 4), with the manufacturers’ RRs, the prevalence of subclinical hypothyroidism decreased significantly in women, from 6.01% to 2.22% (*P* < .0001), when using the new age- and sex-specific RRs. Similarly, prevalence in men decreased from 3.68% to 2.22%. These decreases were mainly due to age-dependent increases in the upper limit of TSH with the new RRs and occurred in all age groups in both sexes, except in 30-39-year-old men.

Using Abbott kits, the prevalence of subclinical hyperthyroidism increased from 1.16% to 1.83% in women and from 1.24% to 2.09% in men when using the new RRs (Tables 1 and 2). These increases were primarily due to an increase in the middle-aged group. Similar results were obtained with the Siemens kits (Tables 3 and 4).

### Other thyroid statuses

Using Abbott kits, the SITSH prevalence significantly increased with the new RRs, from 0.03% to 1.91% (*P* < .0001) in women and 0.02% to 1.86% (*P* < .0001) in men (Tables 1 and 2). These increases might be mainly due to a decrease in the upper limit of FT4 in the age- and sex-specific RRs compared to that in the manufacturers’ RRs. Most increases were observed in 30-69-year-old women and 30-79-year-old men. Using Siemens kits (Tables 3 and 4), no women were identified with SITSH using the manufacturers’ RRs, but this increased to 1.53% when using the new RRs. In men, the prevalence of SITSH increased significantly from 0.11% to 1.40% (*P* < .0001).

We also analyzed the thyroid status of patients with low serum FT4 and normal/reduced TSH levels. With Abbott kits, the percentage of individuals with low FT4 and normal TSH increased from 0.12% to 1.65% (*P* < .0001) and 0.02% to 1.92% (*P* < .0001) in women and men, respectively (Tables 1 and 2), mainly due to more middle-aged individuals. Few cases with low FT4/low TSH levels were identified with either set of RRs. Similarly, with the Siemens kit, the percentage of individuals with low FT4 and normal TSH increased from 0.03% to 0.54% in women (*P* < .0001) and 0.04% to 0.90% in men (*P* < .0001; Tables 3 and 4), primarily among middle-aged individuals. Low FT4 and TSH levels were observed in one woman and one man using both RRs.

### Effect of the new age- and sex- specific reference ranges on the association between thyroid status and metabolic disorders

Since the prevalence of various thyroid statuses changed when using the new age- and sex- specific RRs, the associations between thyroid status and metabolic disorders were also expected to change. We examined the risk of metabolic disorders in subclinical hypothyroidism, subclinical hyperthyroidism, and SITSH, using either the manufacturers’ RRs or the new RRs.

Using Abbott kits with the manufacturer's RRs, none of these thyroid disorders were associated with increased risk of dyslipidemia, hypertension, or diabetes. However, with the new RRs, while subclinical hypothyroidism and subclinical hyperthyroidism remained non-significant risk factors, SITSH was associated with an increased risk of both hypertension (OR 2.00; 95% CI 1.03-3.88) and diabetes (OR 2.65; 95% CI 1.21-5.81) (Table 5).

**Table 5:** Association between thyroid statuses and metabolic diseases using Abbott kits in age- and sex- adjusted logistic regression models

<Dyslipidemia>						
	manufacturer's reference ranges			age- and sex-specific reference ranges		
	Odds ratio	95% CI of OR	*P* value	Odds ratio	95% CI of OR	*P* value
subclinical hypothyroidism	1.51	0.91-2.53	.1128	1.43	0.94-2.19	.0967
subclinical hyperthyroidism	0.74	0.36-1.52	.409	0.89	0.53-1.51	.6752
SITSH	—	—	—	1.10	0.67-1.81	.7048
						

<Hypertension>						
	manufacturer's reference ranges			age- and sex-specific reference ranges		
	Odds ratio	95% CI of OR	*P* value	Odds ratio	95% CI of OR	*P* value
subclinical hypothyroidism	0.60	0.22-1.67	.3326	0.75	0.30-1.85	.5285
subclinical hyperthyroidism	0.96	0.30-3.06	.9399	0.82	0.33-2.03	.6717
SITSH	—	—	—	2.00	1.03-3.88	.**0415**
						

<Diabetes>						
	manufacturer's reference ranges			age- and sex-specific reference ranges		
	Odds ratio	95% CI of OR	*P* value	Odds ratio	95% CI of OR	*P* value
subclinical hypothyroidism	0.89	0.28-2.86	.8399	0.87	0.27-2.76	.808
subclinical hyperthyroidism	1.20	0.29-4.95	.7995	1.28	0.46-3.52	.6343
SITSH	—	—	—	2.65	1.21-5.81	.**0151**

Significance of bold values represents *P* < .05.

Odds ratio was adjusted for age and sex.

-; Odds ratios were not calculable due to zero events.

Abbreviations: OR, odds ratio; CI, confidence interval; SITSH, Syndrome of inappropriate secretion of TSH

Using Siemens kits with the manufacturer's RRs, subclinical hypothyroidism was associated with an increased risk of dyslipidemia (OR 1.21; 95% CI 1.02-1.42) and a decreased risk of hypertension (OR 0.72; 95% CI 0.57-0.91). However, these associations were not observed when subclinical hypothyroidism was defined using the new RRs. Subclinical hyperthyroidism was not associated with any metabolic disorders using either RRs. When using the new RRs, SITSH was associated with an increased risk of hypertension (OR 1.70; 95% CI 1.19-2.44) and diabetes (OR 1.68; 95% CI 1.13-2.51), but not when using the manufacturer's RRs, consistent with the results from Abbott kits (Table 6).

**Table 6:** Association between thyroid statuses and metabolic diseases using Siemens kits in age- and sex-adjusted logistic regression models

<Dyslipidemia>						
	manufacturer's reference ranges			age- and sex-specific reference ranges		
	Odds ratio	95% CI of OR	*P* value	Odds ratio	95% CI of OR	*P* value
subclinical hypothyroidism	1.21	1.02-1.42	.**0252**	1.12	0.89-1.41	.327
subclinical hyperthyroidism	0.90	0.62-1.30	.5697	0.92	0.70-1.23	.5833
SITSH	4.50	0.94-21.52	.0597	1.07	0.80-1.42	.6581

<Hypertension>						
	manufacturer's reference ranges			age- and sex-specific reference ranges		
	Odds ratio	95% CI of OR	*P* value	Odds ratio	95% CI of OR	*P* value
subclinical hypothyroidism	0.72	0.57-0.91	.**0056**	0.76	0.52-1.09	.1327
subclinical hyperthyroidism	0.99	0.57-1.72	.9722	1.23	0.84-1.79	.2813
SITSH	1.02	0.12-8.44	.9829	1.70	1.19-2.44	.**0038**

<Diabetes>						
	manufacturer's reference ranges			age- and sex-specific reference ranges		
	Odds ratio	95% CI of OR	*P* value	Odds ratio	95% CI of OR	*P* value
subclinical hypothyroidism	0.78	0.59-1.03	.0797	0.77	0.50-1.18	.2327
subclinical hyperthyroidism	0.96	0.51-1.82	.9044	1.16	0.75-1.81	.5046
SITSH	—	—	—	1.68	1.13-2.51	.**011**

Significance of bold values represents *P* < .05.

Odds ratio was adjusted for age and sex.

-; Odds ratios were not calculable due to zero events.

Abbreviations: OR, odds ratio; CI, confidence interval; SITSH, Syndrome of inappropriate secretion of TSH

## Discussion

We demonstrated the prevalence of thyroid statuses based on our recently reported new age- and sex-specific serum FT4 and TSH RRs and compared these to those based on the RRs provided by manufacturers of two different kits. We observed an increased prevalence of SITSH and individuals with low FT4/normal TSH levels, and a slightly increased prevalence of subclinical hyperthyroidism using both kits. Additionally, changes in the prevalence of subclinical hypothyroidism differed between the two kits, with an increase observed with the Abbott kits and a decrease with the Siemens kits. These results indicated underdiagnosis of most thyroid statuses when using the current manufacturers’ RRs, while under- and overdiagnosis of subclinical hypothyroidism were kit-dependent. In addition, we assessed the associations between thyroid status and metabolic disorders and found that SITSH, as defined using the new RRs with both Abbott and Siemens kits, increased the risk of hypertension and diabetes mellitus.

We used the middle 95% level ranges obtained from over 8000 individuals to define our new RRs. This indicates that at least 5% of apparently healthy individuals have levels that fall above or below the RR [7]. Thus, our prevalence of abnormal thyroid status was reasonable. According to Abbott, their FT4 RRs are based on the middle 99% rather than the middle 95% of values. The RR-dependent differences in thyroid status prevalence may relate not only to age and sex adjustments but also to differences in how ranges are set. Medical staff and general physicians should be aware that the blood test results might include many false-positive cases, since they are used to screen for a certain disease and to prevent false-negative results, and that there are differences in the RRs established by different kits.

The SITSH prevalence was approximately 1.5% using the new RRs for both Siemens and Abbott kits. These values were higher than those obtained with the manufacturers’ RRs. Resistance to TH (RTH) and TSH-producing pituitary neuroendocrine tumors (PitNET) are well-known causes of SITSH [3, 30]. The incidence of RTH has been reported as one in 40 000 people in Japan [31], and that of TSH-producing PitNET as 2.8 per 1 million people per year in 2010 in Sweden [32]. Therefore, the high prevalence of SITSH might not indicate a higher frequency of RTH and TSH-producing PitNET but rather reflect individuals whose physiological thyroid hormone setpoints were outside the normal reference range, when using the age- and sex-specific RRs. Additionally, the prevalence of patients with low FT4/normal TSH was higher using the new RRs than using the manufacturers’ RRs. Generally, most of the low FT4/normal TSH or low FT4/low TSH indicates central hypothyroidism. The prevalence is reported as one in 20 000–80 000 people [33]. Conversely, pituitary adenomas have been detected in 16.4% of patients undergoing imaging evaluation or pathological autopsy [34]. Previous data showed that approximately 15% of the non-functional pituitary adenomas requiring surgery were associated with central hypothyroidism [35]. Considering these data, there might be cases of non-diagnostic central hypothyroidism without clinical symptoms. Further study is needed to diagnose these relatively rare diseases.

In contrast to the prevalence of most thyroid dysfunctions, which primarily changed in the middle-aged population, the prevalence of subclinical hypothyroidism showed significant changes in not only middle-aged but also older people. This suggests that age adjustments have a greater impact on the diagnoses of subclinical hypothyroidism. Previous studies regarding the prevalence and treatment effects of subclinical hypothyroidism have reported inconsistent results, particularly in older populations [22-27]. These discrepancies may be attributed to using consistent manufacturers’ RRs.

The different thyroid function test kits showed different degrees of change according to age and sex, although all showed similar patterns. This variation is thought to contribute to the differences in the prevalence observed between the kits. The kits used for evaluating thyroid status in each study and hospital should be carefully considered.

Lastly, we investigated the association between thyroid status and metabolic disorders. Notably, SITSH, as defined using the new age- and sex-specific RRs, was significantly associated with an increased risk of hypertension and diabetes, regardless of whether Abbott or Siemens kits were used. While TSH-producing PitNETs are known to present with symptoms of hyperthyroidism [36], and patients with RTH may exhibit palpitations [37], the specific relationship between SITSH and metabolic disorders has remained unclear. Given the higher prevalence of SITSH identified using the new age- and sex- specific RRs compared to that identified using the manufacturers’ RRs, our findings suggested the existence of a previously unrecognized subset of SITSH. Importantly, this atypical thyroid status appears to be associated with cardiovascular risk factors. Most individuals with SITSH were middle-aged, highlighting the potential clinical importance of early detection of this thyroid status to help prevent future cardiovascular diseases.

This study had some limitations. Ethnicity may influence serum TSH and TH levels [10, 38-41]. Since more than 95% of our participants were Japanese, the effects of ethnicity and place of residence were likely minimal. Second, unlike a population-based cohort study, this study included participants undergoing health checkups; therefore, some bias was present in terms of participants’ economic status. Third, this study was performed in Japan, which is generally considered an iodine-sufficient country. However, even in iodine-sufficient regions, functional iodine deficiency may occur if iodine uptake into thyroid cells is inhibited by environmental factors such as perchlorate or thiocyanate [42, 43]. In addition, iodine intake levels and environmental exposure vary among countries, and these factors should be taken into account [38, 44]. These results also need to be validated in other geographic and ethnic groups, given that the prevalence of autoimmune diseases may also differ. Fourth, the number of younger and older participants may have been insufficient for statistical analyses.

In conclusion, this study clearly demonstrated the need for age- and sex-specific RRs for serum FT4 and TSH levels for thyroid status diagnosis. Over- and under-diagnosis of subclinical hypothyroidism and hyperthyroidism should be avoided to ensure appropriate therapy. SITSH and other thyroid conditions may be more common than are currently diagnosed. Moreover, SITSH defined using age- and sex-specific RRs appeared to be associated with metabolic disorders. Future studies using age- and sex-specific RRs may be required to reevaluate the prevalence and effectiveness of treatments for these conditions.

## Data Availability

Restrictions apply to the availability of some or all data generated or analyzed during this study to preserve patient confidentiality or because they were used under license. The corresponding author will, on request, detail the restrictions and any conditions under which access to some data may be provided.
